# Glutamic Acid Decarboxylase (GAD-65) Autoimmunity Associated With Profound Daytime Hypersomnia, Nighttime Insomnia, Mild Autonomic Neuropathy and Axonal Sensori-Motor Polyneuropathy: A Case Report on a New Phenotype

**DOI:** 10.7759/cureus.11112

**Published:** 2020-10-23

**Authors:** Hassan Kesserwani

**Affiliations:** 1 Neurology, Flowers Medical Group, Dothan, USA

**Keywords:** auto immune, anti-gad

## Abstract

We describe the case of a 74-year-old fit and healthy man who developed a profound sleep disorder characterized by mid-day hypersomnia and debilitating insomnia. A wide range of therapies, including a large number of stimulants and hypnotics with multiple different mechanisms of action, failed to improve his condition. Trials with oral prosthetic devices and a wide range of face masks with positive pressure assistance and multiple continuous positive airway pressure (CPAP) titration studies failed to help. Along with his sleep disorder, our patient developed a slowly evolving axonal sensorimotor polyneuropathy with a subtle autonomic neuropathy. Due to the latter two conditions, a comprehensive paraneoplastic panel was obtained and revealed extremely high titer glutamic acid decarboxylase (GAD-65) autoantibodies. This was confirmed by three independent laboratories and by cerebrospinal fluid staining of rat hippocampus, revealing the classic tram-track lines along the dentate gyrus. Our patient was treated empirically with intravenous immunoglobulin. We believe that our case reveals a unique syndrome related to GAD-65 autoantibodies and adds to the growing list of GAD-65 associated diseases. This case is particularly provocative as it raises the idea to check for GAD-65 autoimmunity in patients who suffer from a profound sleep disorder resistant to conventional treatment.

## Introduction

Glutamic acid decarboxylase (GAD) is a neuronal enzyme involved in gamma-aminobutyric acid (GABA) synthesis by decarboxylation of glutamic acid to GABA, an inhibitory neurotransmitter. Antibodies directed against the 65 kilodalton (kd) isoform of GAD, GAD-65, are seen in stiff-person syndrome, autoimmune cerebellar ataxia, brainstem encephalitis, autoimmune epilepsy, neuromyelitis optica, autoimmune transverse myelopathies, myasthenia gravis, Lambert-Eaton myasthenic syndrome, and autonomic neuropathies. GAD-65 antibody is a beta-cell pancreatic islet antibody and a serological marker for susceptibility to type I diabetes, thyroid diseases (Grave's disease, Hashimoto's thyroiditis), pernicious anemia, Addison's disease, and vitiligo. Low titer antibodies to GAD-65 are found in eight percent of healthy subjects older than age 50 [1].

It is important to distinguish between low and high titer antibodies. High titers, greater than 0.03 nanomoles per liter (nmol/L), are found in stiff-person syndrome (93%), autoimmune cerebellar ataxia, autoimmune encephalo-myelopathies, and in autoimmune type I diabetes. Low titers, less than 0.02 nmol/L, are found in the serum of type II diabetic patients (less than five percent), myasthenia gravis (25%), the Lambert-Eaton myasthenic syndrome, and type I diabetes without other endocrine involvement [2]. 

The serologic test is a radio-immunoassay test. The patient's serum is incubated with pellets of Iodine-125 labeled recombinant human GAD-65 protein. Anti-human immunoglobulin G (IgG) and immunoglobulin M (IgM) antibodies are then added to the mixture; an immunoprecipitate is formed. The precipitated immune complexes are then washed, after which specific antibodies are detected by gamma-ray emission from the pellets. As noted above, low titers are usually less than 0.02 nmol/L and high titers greater than 0.03 nmol/L [2]. This distinction is important as low titers are frequently seen in healthy individuals and less serious diseases.

GAD-65 is located intracellularly and is not usually accessible to antibodies. The hypothesis is that GAD-65 transiently appears on the membrane of the synaptic cleft during neurotransmission [3]. The mechanism of injury is thought to be due to a T-cell mediated paraneoplastic process [4]. Furthermore, GAD-65 antibodies are not pathogenic in animal models by transfusing them with GAD-65 auto-antibodies, explaining the limited efficacy of immunotherapy. Partial response to immunotherapy is the rule, and dual immunotherapy is frequently necessary [5]. Cerebrospinal fluid intrathecal synthesis of GAD-65 antibodies demonstrated by in-vitro binding to rat dentate gyrus and oligoclonal band testing should be performed in patients with central nervous system disease who carry high serum levels of GAD-65 auto-antibodies [6].

We present the case of a 74-year-old highly productive professional who, over the course of 12 years, has developed the insidious onset of a treatment-resistant profound sleep disorder characterized by daytime hypersomnia and nighttime insomnia. This was associated with the development of a slowly progressive axonal sensorimotor polyneuropathy with impairment of balance and a subtle autonomic neuropathy characterized by blood pressure volatility, intermittent diaphoresis, and gastrointestinal distress. Our patient developed extremely high serum GAD-65 auto-antibodies, which were also detected in the cerebrospinal fluid by immunostaining of a rat dentate gyrus with the typical tram-track outline. We believe that this phenotype has never been described before. In the discussion section, we review the literature of GAD-65 phenotypes that are similar to our patient's presentation, discuss the mechanisms of neuronal injury, and briefly overview immunotherapy.

## Case presentation

We present a 74-year-old fit, healthy, highly intelligent, and productive professional who, for over 12 years and especially over the last year, has developed progressive symptoms involving multiple domains. Specifically, he has developed exhaustion around noontime, after a morning of a high caseload of activities. He was always able to do this with ease, but especially over the last six months, the urge to sleep has become irresistible at mid-day. He has also noted mental fatigue following intense intellectual activities, such as in business meetings. His manual skills remain sharp and acute. For many years he has had non-bothersome mild tingling and burning in the feet with mild acral swelling. He is able to get out of a chair and climb stairs with ease but over the last years has noted some balance issues. After meals, he feels the urge to have a bowel motion, but with normal bowel frequency. At times he has unprovoked night-time intense sweats. On one such occasion, he felt weak, and his blood pressure spiked, after which he ended up in the emergency room with spontaneous resolution of symptoms. He has also been struggling with poor sleep for the last eight years. A sleep study revealed mild obstructive sleep apnea with an apnea-hypopnea index (AHI) of 6. This becomes moderate during rapid eye movement (REM) sleep with an AHI of 25. He was tried on auto-pap (positive airway pressure) from 6 to 10 centimeters (cm) of pressure, without much help. His Epworth Sleepiness score is 10. He wakes up about four to five times nightly for five to fifteen minutes and has tried many sleep agents without relief. He may fall asleep easily but has difficulty maintaining sleep. He swims and exercises daily, reads avidly, retires to bed around 10 pm nightly, and wakes up at 6 am. A trial of finasteride 5 milligrams (mg) daily has helped his recent development of bladder urgency and frequency. He denies dry eyes or dry mouth.

The recent development of mild hypertension has improved with ramipril 5mg daily. Consultation with two sleep specialists and multiple sleep studies with trials of multiple brands of face masks has not helped his sleep hygiene. Hypnotic agents, including melatonin, ramelteon, zolpidem, eszopiclone, trazodone, and suvorexant, have met with mixed success due to lack of efficacy, tolerance, and the morning hangover effect of some of these medications. Trials with modafinil and armodafinil have not helped his fatigue, but the recent introduction of pitolisant at a 4.45 mg daily dose has had modest effects. He lives a regimented lifestyle of hard work, business meetings, regular exercise, and swimming. He is a lifetime non-smoker.

His past medical history includes paroxysmal atrial fibrillation, which led to a small cortical left posterior parietal infarct from which he made a complete recovery. He had a pacemaker installed for a history of bradycardia and a left atrial appendage occlusion for atrial fibrillation and continues with the oral anticoagulant apixaban 5 mg twice daily due to his prior stroke history. The recent development of testosterone deficiency has been corrected with hormone replacement therapy. However, through his astute observation, he notes that when his testosterone levels fall, he develops night sweats and nocturnal leg cramps that resolve with adequate testosterone replacement.

Medications include low dose ramipril 10 mg daily, apixaban 5 mg twice daily, finasteride 5mg daily, testosterone patch 4 mg per 24 hours, and levothyroxine 50 micrograms daily. 

Family history is significant for maternal atrial fibrillation and a brother who has a mild peripheral neuropathy. 

On examination, his blood pressure was 110/60, with a pulse of 60. His height is six feet, four inches with a weight of 190 pounds and a body-mass index (BMI) of 23.1. Cardiac auscultation was negative for a murmur and carotid bruits were absent. Cognitively, he is sagacious and highly intelligent. He was well prepared with a detailed summary of his complex history. His visuomotor, limb-kinetic, and ideomotor praxes were fluid and fluent. His gait cadence was swift with a normal arm swing. Tandem walking was performed cautiously. Romberg's sign was absent, and he was able to keep his eyes closed for at least 15 seconds. Heel and toe walking was done carefully. He can stand on either foot independently and with relative ease for at least ten seconds. Repetitive fine finger motion with sequence motion was normal. No dysmetria was noted with finger to nose pointing or heel to shin maneuver bilaterally. No rest tremor, postural or kinetic tremor was noted. No bradykinesia was noted either.

Cranial nerve examination was entirely normal. The blinking frequency was normal, at least 12 per minute. Pertinent negative findings were an absent Adie's pupil or Horner syndrome. No peri-oral twitching was noted.

Power examination in both upper and lower limbs was graded 5/5 proximally and distally, using the Medical Research Council (MRC) grading. Deep tendon reflexes were lively in the arms and legs, except for absent ankle jerks bilaterally. Sensory examination revealed hypesthesia to vibration in the feet with a 128 Hertz tuning fork. Joint position sense in the toes, touch, pressure, and pin-prick was preserved. There was a diminished cold perception in the feet. The extensor digitorum brevis was atrophied in both feet.

Skin examination revealed prominent vitiligo patches in the lower legs and forearms. No pitting edema was noted. 

His laboratory studies are listed in Table 1.

**Table 1:** Patient's laboratory values Note extremely high GAD 65 antibodies, hypothyroidism, high anti-thyroglobulin antibodies, high calcium channel antibody P/Q type nmol/L - nanomole per liter, IU/ml - international units/milliliter, CRMP - collapsin response mediator protein, IgG - immunoglobulin G, pg/ml - picogram per milliliter, ANA - antinuclear antibody, TSH - thyroid-stimulating hormone, GAD - glutamic acid decarboxylase, DNA - deoxyribonucleic acid, H - high, U/L - units per liter, mg/dL - milligram per deciliter

Test	Result	Normal value	Test	Result	Normal value
Anti-neuronal nuclear antibodies, Type 1, 2, 3	Negative	< 1: 240	Calcium channel antibody P/Q type antibody	0.04 (H)	< 0.02 nmol/L
Anti-glial nuclear antibody, Type 1	Negative	< :1:240	Thyroid-stimulating hormone (TSH)	5.120 (H)	0.450-4.500 IU/ml
Purkinje cell cytoplasmic antibody, Type 1, 2, 3	Negative	< 1: 240	Anti-thyroglobulin antibodies	1.9 (H)	< 1.0 IU/ml
Amphiphysin antibody	Negative	< 1: 240	Glutamic acid decarboxylase (GAD) 65 antibody	216 (H)	< 0.02 nmol/L
CRMP-5 IgG	Negative	< 1: 240	Thyroglobulin antibody	1.3 (H)	< 0.9 IU/ml
Striational antibodies (striated muscle)	Negative	< :1: 220	Anti-thyroid peroxidase antibody	3.8	< 9 IU/ml
Calcium channel antibody, N-type	0.00	< 0.03 nmol/L	Thyroid peroxidase antibody	11	< 34 IU/ml
Acetylcholine receptor ganglionic neuronal antibody	0.00	< 0.02	Vitamin B12	1273	> 232 pg/ml
Neuronal voltage-gated potassium channel antibody	0.00	< 0.02	Anti-double-stranded DNA antibody	< 1	> 9 IU/ml
Creatine kinase	140	< 331 U/L	Fasting glucose	90	< 125 mg/d;L
ANA comprehensive panel	Negative		Immunofixation electrophoresis	No monoclonal protein	
Hemoglobin A1C	5.3%	< 6.4 %			

**Figure 1:**
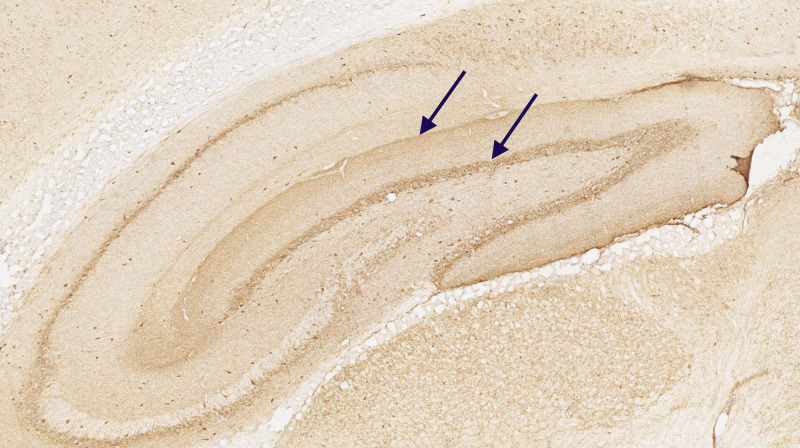
Immunostaining of rat hippocampus with GAD-65 antibodies: tram-track staining of dentate gyrus (blue arrows) Histology slide GAD-65 - glutamate acid decarboxylase

Table 1 displays an extremely high GAD-65 antibody, high calcium channel antibody P/Q type (twice normal), anti-thyroglobulin antibodies, and mild hypothyroidism. A repeat calcium channel (P/Q) antibody at another institution was negative, testifying to the wide variability of low titer auto-antibodies. A repeat GAD-65 antibody at another institution by enzyme-linked immunosorbent assay (ELISA) with serial dilutions revealed an extremely high GAD-65 auto-antibody level of > 10,000 International Units per milli-liter (IU/ml; normal < 250 IU/ml). The cerebrospinal fluid analysis revealed normal glucose and protein levels, with the absence of pleocytosis. The immunoglobulin G index (IgG index) was normal. Oligoclonal banding was present in cerebrospinal fluid (CSF). Immunostaining of rat hippocampus with GAD-65 auto-antibody revealed the typical pattern of tram-track staining of the dentate gyrus (Figure 1).

A visit to an endocrinologist led to a diagnosis of low grade Hashimoto's thyroiditis, which is being treated with low dose thyroid hormone. He has undergone an extensive evaluation including a computed axial tomography (CAT) scan of the chest, abdomen, and pelvis with and without contrast, with no significant findings, except for a small right lower lung pulmonary nodule which has been stable for many years and is being monitored by a pulmonologist. A urologic evaluation revealed mild prostatic hypertrophy, for which he receives finasteride, and testosterone deficiency for which he is on a testosterone patch, as noted above. For his occasional orthostatic intolerance, a standard 70-degree head-up tilt table test was done (Table 2). There was a mild drop in systolic blood pressure upon initial tilting, which auto-corrected. He was asymptomatic throughout the tilt-table test.

**Table 2:** Tilt table test with 70-degree head-up elevation: initial mild drop in systolic blood pressure which auto-corrected; patient asymptomatic throughout the study

	Supine	0 minutes	1 minute	5 minutes	10 minutes	20 minutes
Blood pressure	110/71	93/61	94/57	98/62	104/65	103/68
Heart rate	60	62	61	60	60	62

A nerve conduction study/electromyography (NCS/EMG) revealed bilateral absent peroneal and sural sensory amplitudes with absent bilateral peroneal and tibial motor amplitudes, consistent with a severe sensorimotor polyneuropathy. The electromyographic study revealed moderately severe chronic distal denervation consistent with a chronic length-dependent polyneuropathy. Due to its severity, we could not establish if the polyneuropathy was axonal or demyelinating. However, a prior study twelve years earlier determined that this polyneuropathy was axonal. A magnetic resonance imaging (MRI) of the brain revealed encephalomalacia of the posterior right parietal cortex consistent with a known old embolic ischemic infarct with no evidence of limbic encephalitis. 

The extremely high serum GAD-65 antibodies, typical tram-track staining of rat dentate gyrus with CSF GAD-65 autoantibodies, and the cluster of symptoms related to the peripheral neuropathy, autonomic neuropathy, and the profound sleep disorder, in our opinion, constitutes a hitherto undefined syndrome associated with GAD-65 autoantibody. We believe this syndrome is probably underappreciated as peripheral neuropathies, autonomic neuropathies, and sleep disorders are all relatively common. However, a profound sleep disorder characterized by daytime hypersomnia, nighttime insomnia that is resistant to conventional treatment should prompt testing for GAD-65 autoantibody. This, in combination with a slowly evolving cryptic polyneuropathy, with no obvious cause, and a subtle autonomic neuropathy with mild blood pressure volatility and gastro-intestinal distress, should also raise the alarm. Our patient was infused with intravenous immunoglobulin (IVIg) 1 gram per kilogram (g/kg) daily for two consecutive days. The plan is to repeat this infusion monthly for a total of three months, repeat GAD-65 auto-antibodies after three months and longitudinally monitor his progress. Should he not respond, we will consider intravenous rituximab.

## Discussion

Paraneoplastic encephalitic syndromes display antibodies directed at intracellular antigens with neuro-degeneration mediated by T cell cytotoxicity. The non-paraneoplastic, autoimmune encephalitic syndromes display antibodies against both neural surface antigens (such as the voltage-gated potassium channel-complexes and N-methyl-D-aspartate (NMDA) receptors) and intracellular antigens (such as GAD-65). The difference in mechanisms of neural injury is listed below (Table 3) [4].

**Table 3:** Immune-mediated pathology comparison between the paraneoplastic and the non-paraneoplastic group T - thymic, B - bursa, CD - cluster of differentiation

Paraneoplastic syndrome	Non-paraneoplastic syndrome / autoimmune syndrome
Intracellular antigens	Both intracellular and extracellular
T-cell mediated	Less T-cell mediated and more B-cell mediated
More cytotoxic T-cell adhesion to neurons with cell loss	Less cytotoxic T-cell adhesion to neurons with cell loss
Higher CD8/CD3 ratio	Lower CD8/CD3 ratio

Of note, the findings of a higher CD8/CD3 ratio and T-cell adhesion to neurons with neuron loss was less severe in the GAD-65 group. Furthermore, a complement (C9) deposition on neurons associated with neuronal cell death was found only in the voltage-gated potassium channel-complex antibody subgroup. Finally, the NMDA receptors antibody group showed no evidence of antibody or complement-mediated attack, and there was an absence of a definite neuronal pathology [4].

Sleep disorders and mild autonomic neuropathies are both prevalent. But a profound disorder of sleep resistant to treatment and a mild autonomic neuropathy associated with extremely high GAD-65 antibodies has not been described. There are rare reports of dysautonomia and autonomic neuropathies associated with anti-GAD antibodies [7]. The two patients of Feliccia et al. showed a reasonable response to steroids in one patient, and they emphasize the need to look for the intrathecal synthesis of antibodies [8]. The Mayo Clinic study of Mckeon et al. does not alert us to the prevalence of autonomic neuropathies [9].

There are very rare reports of profound gastrointestinal symptoms, a case of chronic intestinal pseudo-obstruction, and rare cases of achalasia [10, 11].

Silber et al. and Iranzo et al. provide authoritative accounts of autoimmune associated sleep disorders, but GAD-65 autoimmunity is not mentioned [12, 13]. There are rare reports of profound insomnia associated with high titer GAD-65 antibodies in a patient with the Klein Levin syndrome and another patient with voltage-gated potassium channel antibodies [14, 15].

These findings are summarized below (Table 4).

**Table 4:** Summary of GAD-65 autoimmunity studies associated with profound sleep disorders or autonomic dysfunction

Study	Disorder	Other associations
Achour NA, et al., 2018, [7]	Dysautonomia	Limbic encephalitis
Fileccia E, et al., 2016, [8]	Small fiber neuropathy	
Maier A, et al., 2015, [10]	Pseudo-obstruction of bowel	Limb stiffness
Kraichely R, et al., 2010, [11]	Achalasia	
Rout UK, et al., 2014, [14]	Profound hypersomnia	Klein Levin syndrome
SabharwaL P, et al., 2016, [15]	Profound insomnia	Voltage-gated potassium channel antibodies

Only 4-6% of patients with GAD-65 positive stiff-person syndrome have associated malignant tumors; non-small cell lung cancer, pancreatic neuroendocrine tumors, thymomas, breast, thyroid, renal, and colon cancer. The association with cancer is more common in cerebellar ataxia and limbic encephalitis, 9-26% [16, 17]. Interestingly, GAD-65 autoimmunity is almost never reported in association with other paraneoplastic neuronal auto-antibodies [18]. Patients with neurological syndromes associated with GAD-65 auto-antibody usually have a personal or familial history of autoimmunity, including type I diabetes mellitus, thyroiditis, pernicious anemia, coeliac disease, vitiligo, and the presence of various auto-antibodies including anti-thyroperoxidase, anti-thyroglobulin, anti-parietal cells, and anti-gliadin antibodies [19].

Muñoz-Lopetegi et al. report a retrospective study of 56 patients' clinical profile with high and low-titer GAD-65 auto-antibodies. The take-home message is that 70% of patients responded to immunotherapy, most commonly IVIg, and intravenous methylprednisolone, with a concomitant drop in GAD-65 auto-antibodies in the vast majority of patients. Relapses were common after discontinuation of therapy. Less common treatments included plasma exchange, rituximab, and cyclophosphamide. The typical phenotypes of the high titer subgroup included stiff-person syndrome, cerebellar ataxia, epileptic syndromes, and limbic encephalitis. The lower titer subgroups manifested other rarer syndromes [5].

## Conclusions

Our case describes a hitherto undefined GAD-65 phenotype. We unequivocally demonstrated very high titers of serum and CSF GAD-65 auto-antibodies. The triad of a severe sleep disorder, daytime hypersomnia, and nighttime insomnia, resistant to conventional treatment plus an axonal sensorimotor polyneuropathy and autonomic neuropathy, constitutes a new phenotype associated with GAD-65 autoimmunity. An extensive literature search has failed to reveal a similar syndrome. A word of caution - these three constituent diseases are very common in the population, and it is likely that we are not screening enough patients for GAD-65 autoimmunity. Our case report opens up fertile grounds for further research in GAD-65 autoimmunity and raises the profound idea to check treatment-resistant sleep disorder patients for GAD-65 autoimmunity.

## References

[1] Dade M, Berzero G, Izquierdo C (2020). Neurological syndromes associated with Anti-GAD antibodies. Int J Mol Sci.

[2] Walikonis JE, Lennon VA (1998). Radioimmunassay for glutamic acid decarboxylase [GAD65] autoantibodies as a diagnostic aid for stiff-man syndrome and a correlate of susceptibility to type 1 diabetes mellitus. Mayo Clin Proc.

[3] Dalakas MC (2013). Progress and stiff challenges in understanding the role of GAD-antibodies in stiff-person syndrome. Exp Neurol.

[4] Bien CG, Vincent A, Barnett MH (2012). Immunopathology of autoantibody-associated encephalitides: clues for pathogenesis. Brain.

[5] Munoz-Lopetegi A, de Bruijn M, Boukhrissi S (2020). Neurologic syndromes related to anti-GAD65 Clinical and serologic response to treatment. Neurol Neuroimmunol Neuroinflamm.

[6] Graus F, Saiz A, Dalmau J (2020). GAD antibodies in neurological disorders — insights and challenges. Nat Rev Neurol.

[7] Achour NA, Younes TB, Rebai I, Ahmed MB, Kraoua I, Youssef-Turki IB (2018). Severe dysautonomia as a main feature of anti-GAD encephalitis: report of a paediatric case and literature review. Eur J Paediatr Neurol.

[8] Fileccia E, Rinaldi R, Liguiri R (2016). Post-ganglionic autonomic neuropathy associated with anti-glutamic acid decarboxylase antibodies. Clin Auton Res.

[9] Mckeon MB, Tracy JA (2017). GAD65 neurological autoimmunity. Muscle Nerve.

[10] Maier A, Mannartz V, Wasmuth H (2105). GAD Antibodies as key link between chronic intestinal pseudoobstruction, autonomic neuropathy, and limb stiffness in a nondiabetic patient: a CARE-compliant case report and review of the literature. Medicine.

[11] Kraichely RE, Farrugia G, Pittock SJ, Castell DO, Lennon VA (2010). Neural autoantibody profile of primary achalasia. Dig Dis Sci.

[12] Iranzo A (2020). Sleep and neurological autoimmune diseases. Neuropsychopharmacology.

[13] Silber M (2016). Autoimmune sleep disorders. Clin Neurol.

[14] Rout UK, Michener MS, Dhossche DM (2014). GAD65 autoantibodies in Kleine-Levin syndrome. J Neuropsychiatry Clin Neurosci.

[15] Sabharwal P, Mahmoudi M, Berberi N (2016). A Case of Recurrent Insomnia: Extending the Spectrum of Autoimmune Encephalitis. J Clin Sleep Med.

[16] McKeon A, Robinson MT, McEvoy KM (2012). Stiff-man syndrome and variants: Clinical course, treatments, and outcomes. Arch. Neurol.

[17] Ariño H, Höftberger R, Gresa-Arribas N (2015). Paraneoplastic neurological syndromes and glutamic acid decarboxylase antibodies. JAMA Neurol.

[18] Pittock SJ, Yoshikawa H, Ahlskog JE (2006). Glutamic acid decarboxylase autoimmunity with brainstem, extrapyramidal, and spinal cord dysfunction. Mayo Clin Proc.

[19] Gresa-Arribas N, Ariño H, Martínez-Hernández E (2015). Antibodies to inhibitory synaptic proteins in neurological syndromes associated with glutamic acid decarboxylase autoimmunity. PLoS ONE.

